# Mapping epistatic quantitative trait loci

**DOI:** 10.1186/s12863-014-0112-9

**Published:** 2014-11-04

**Authors:** Cecelia Laurie, Shengchu Wang, Luciana Aparecida Carlini-Garcia, Zhao-Bang Zeng

**Affiliations:** Department of Mathematics, University of Alabama, Tuscaloosa AL, USA; Department of Biostatistics, University of Washington, Seattle WA, USA; Bioinformatics Research Center, Department of Statistics, North Carolina State University, Raleigh NC, 27695-7566 USA; Instituto Agronômico de Campinas, Centro de Grãos e Fibras, Campinas SP, Brazil; APTA Regional, Pólo Centro Sul, Piracicaba SP, Brazil; Department of Biological Sciences, North Carolina State University, Raleigh NC, USA

**Keywords:** Quantitative trait loci, Epistasis, Model selection, Sequential search

## Abstract

**Background:**

How to map quantitative trait loci (QTL) with epistasis efficiently and reliably has been a persistent problem for QTL mapping analysis. There are a number of difficulties for studying epistatic QTL. Linkage can impose a significant challenge for finding epistatic QTL reliably. If multiple QTL are in linkage and have interactions, searching for QTL can become a very delicate issue. A commonly used strategy that performs a two-dimensional genome scan to search for a pair of QTL with epistasis can suffer from low statistical power and also may lead to false identification due to complex linkage disequilibrium and interaction patterns.

**Results:**

To tackle the problem of complex interaction of multiple QTL with linkage, we developed a three-stage search strategy. In the first stage, main effect QTL are searched and mapped. In the second stage, epistatic QTL that interact significantly with other identified QTL are searched. In the third stage, new epistatic QTL are searched in pairs. This strategy is based on the consideration that most genetic variance is due to the main effects of QTL. Thus by first mapping those main-effect QTL, the statistical power for the second and third stages of analysis for mapping epistatic QTL can be maximized. The search for main effect QTL is robust and does not bias the search for epistatic QTL due to a genetic property associated with the orthogonal genetic model that the additive and additive by additive variances are independent despite of linkage. The model search criterion is empirically and dynamically evaluated by using a score-statistic based resampling procedure. We demonstrate through simulations that the method has good power and low false positive in the identification of QTL and epistasis.

**Conclusion:**

This method provides an effective and powerful solution to map multiple QTL with complex epistatic pattern. The method has been implemented in the user-friendly computer software Windows QTL Cartographer. This will greatly facilitate the application of the method for QTL mapping data analysis.

**Electronic supplementary material:**

The online version of this article (doi:10.1186/s12863-014-0112-9) contains supplementary material, which is available to authorized users.

## Background

Epistasis is an important, yet difficult, question to study in quantitative genetics. The difficulty mainly lies in the biological complexity of epistasis of quantitative trait loci (QTL). There could be interactions among multiple QTL. Some interacting QTL could be in linkage, and some may even have little or no main effect. Relatively small sample size in many QTL mapping experiments adds to the challenge. All these complexities post serious challenges for mapping QTL epistasis accurately and reliably.

There are several strategies proposed in the literature to tackle QTL epistasis. One strategy is to extend an one-dimentional genome scan to a two-dimensional genome scan to search for QTL with or without epistasis in pairs [1], or to a multi-dimensional genome scan to search for the best fitting model [2]. These methods are generally targeted to search for a few QTL, and tend to work well in relatively simpler genetic situations. In more complex situations with a moderate number of interacting QTL, these methods can be inadequate. A potential problem for a simple two-dimensional genome scan is that the searched signals for “epistasis” could be due to other QTL effects either in linkage or more complex epistatic pattern creating a bias in the inference of QTL and epistasis. The statistical power for finding epistasis also can be relatively lower if other segregating QTL effects are not controlled in the model. For a multi-dimensional search, beside the increased computational demand, a key issue is an appropriate evaluation of the signal to noise ratio in the model selection. Manichaikul *et al.* [3] proposed a model search based on identifying sequentially a pair of interacting QTL with model selection based on a penalized LOD score with separate penalties on main and interaction effects to address the signal-to-noise ratio. However, a two- or multi-dimensional search for QTL epistasis is not necessarily more powerful, and actually can be less powerful, than other alternative approaches, such as sequential search [4]. Bayesian approaches have also been proposed, see [5-7] among others. Several of these approaches use reversible jump algorithm to account for varying size of the model space. Yi [7] avoids this by restricting to a single fixed dimension composite space. This requires an estimate of the upper bound on the number of QTL and prior specifications of the inclusion probabilities of main and interaction effects based on prior expected numbers of main-effect and epistatic QTL. This requires some initial computation using other methods and raises the issue of sensitivity to priors.

In this study, we report an approach that has the capacity to map multiple QTL with complex epistasis and has a clear and efficient procedure to evaluate the signal-to-noise ratio to guide the model selection. The approach breaks the search problem into several stages: the first stage is targeted to search for QTL that have significant main effects; the second stage for QTL that interact significantly with the main effect QTL, and the third stage for additional epistatic QTL that may not have significant main effects. Similar ideas of seaching QTL main and epistatic effects in stages have also been proposed previously by [8-10] in different forms.

There are good justifications for this sequential process. In the examination of genetic models with linkage and epistasis, Wang and Zeng [11] showed that the main and epistatic effects of QTL can be partitioned into separate components that are independent even with linkage in a backcross population. This suggests that the main and epistatic effects of QTL can be searched for at different stages without significant risk of introducing bias for later search stages. Since most genetic variation is due to main effects, it would be prudent to search for main effect QTL first. It is relatively easier and robust to map main effect QTL. After main effect QTL are mapped that explain the majority of genetic variation, the statistical power in the subsequent search for epistatic QTL is increased, as the residual variation of the statistical model is reduced. Also computationally, this sequential strategy is much more efficient than a multiple dimensional search.

Critically important to any search procedure is an appropriate and efficient evaluation of sampling variation of a test statistic. An effective procedure must account for issues of multiple testing across the genome and the fact that many factors (such as genome size, genetic map density, informativeness of markers, and proportion of missing data) can affect the distribution of the test statistic. Using data-based empirical distributions is the most effective way to take these factors into account. One widely accepted approach is the use of sequential permutation tests in the context of a nested sequence of models [12,13]. These involve a high computational burden and have drawbacks that might limit robustness in the multiple-QTL model. Broman and Speed [14] proposed a modified BIC criterion using a permutation-based factor that increases the penalty on the size of the model over the standard BIC criterion. They propose a factor that corresponds to a genome-wide LOD threshold for interval mapping for finding one QTL. This approach was generalized in [3] by imposing penalties based on number of QTL and number of interaction terms where the interaction penalty factor is based on a permutation test using a two-dimensional scan for two interacting QTL assuming the null hypothesis of no QTL.

Ideally one would like to use a permutation-type test for the given model at hand at each step of admitting new QTL or interactions to the model. A main ingredient of our proposed method is the adoption and extension of the score-statistic-based re-sampling procedure of [15]. The score statistic re-sampling has several advantages. The procedure is data and model based and takes the sampling variation in the relevant search space into account. It is more computationally feasible than permutation procedures while providing the advantages of using empirical test statistic distributions. It is also important to note that it is adaptable to the varying models and null hypotheses arising in different stages.

We report the details of this sequential search strategy and a simulation study to examine its properties and performance. The simulation study includes genetic architecture reflecting the complexity of epistatic interactions along with realistic sample size and heritablity. We demonstrate that the method can identify QTL with epistasis with good power and low false positive. We also address the issue of the precision of the position estimates for the QTL. The procedure has been implemented in Windows QTL Cartographer [16] which is available at http://statgen.ncsu.edu/qtlcart/WQTLCart.htm.

## Methods

### Model

We use a multiple interval mapping (MIM) model. Assuming *m* putative QTL in a backcross population, the MIM model is defined by

$$y_{i} = \mu + \sum\limits_{k=1}^{m} a_{k}x_{ik} + \sum\limits_{k \ne l \in \{1,\ldots, m\}}\delta_{kl} \gamma_{kl}x_{ik}x_{il} + \varepsilon_{i}. $$

Here *y*_*i*_ is the phenotypic trait value for individual *i* for *i*=1,…,*n* while *x*_*ik*_ is a coded variable denoting the genotype of putative QTL *k* using the Cockerham scale:

$$x_{ik}= \left\{\begin{array}{rc} \frac{1}{2} & \text{if genotype of QTL}\, \, k\, \, \text{for individual}\, \, i\, \, \text{is homozygous}\\ -\frac{1}{2}& \text{if genotype of QTL}\, \, k\, \, \text{for individual}\, \, i\, \, \text{is heterozygous} \end{array} \right. $$

The variable *x*_*ik*_ is unobserved, but its conditional probability can be analyzed given observed marker genotypes and specific positions for the QTL [17]. Parameters include the mean (*μ*), the marginal effects of the putative QTL (*a*_*k*_’s), the epistatic effects of QTL (*γ*_*kl*_’s), and the variance (*σ*^2^) of the residual effects *ε*_*i*_, assumed to be normally distributed with mean zero. We use only a subset of all QTL pairs where the indicator variable *δ*_*kl*_ takes the value one if QTL *k* and QTL *l* interact and takes the value zero otherwise.

Since the genotypes of an individual at the putative QTL are not usually observed (but marker genotypes are), the model contains missing data and thus the likelihood function of the data given the model is a mixture of 2^*m*^ normal distributions. We assume there is no crossover interference and also that double recombination events within an interval between markers are very rare and can be ignored. The mixing proportions are the probabilities of each multi-locus genotype conditioned on marker data [17]. When *m* is large, the number of possible mixture components (QTL genotypes) can become very large and hence many of the mixing proportions can be very small. In a practical implementation of MIM, a selection procedure is adopted to choose a subset of ‘significant’ mixing proportions for evaluation and normalize so that the sum of the probabilities equals 1. A conditional expectation/maximization (EM) algorithm is used to estimate parameters. The MIM model was developed in a series of papers [17-20].

### Model search and model selection

The model selection is in three stages: (1) search for main effect QTL along with interaction effects among the main effect QTL, (2) search for epistatic QTL with significant interaction with a main effect QTL, and (3) two-dimensional search for other epistatic QTL pairs (where neither QTL has significant main effect). At the end of each stage, we optimize the model using backward elimination and optimization of position.

A key ingredient in model selection is choosing appropriate test statistics and corresponding threshold values. We extend the use of the score statistic re-sampling procedure introduced in [15]. The score statistic is an approximation to the full likelihood ratio test. The score statistic can be approximated by a sum of independent random vectors, which enables a re-sampling approach, weighting each term by a random normal variable. The distribution of the re-sampled statistics are used to derive genome-wide thresholds. The stages involve forward searches for positions of QTL to add to the model, testing for admitting interaction parameters, and backward elimination phases. The forward searches for positions along the genome require a genome-wide threshold for an empirical distribution of maximum score statistics. Testing for admitting or deleting additional interaction parameters given that QTL are identified or deleting QTL in the model do not involve a genome search and thus we use a pointwise threshold for testing of significance. (See Additional file 1 for further details.)

We first describe some common parameters and terminology used. For determining threshold values, we use 0.05 (95*%*) as the significance level. For a given identified QTL, we define its *QTL window* as the set of positions within 10 cM to the right or left of the QTL position or until a marker is reached. The genome-wide searches are done using a grid of 1.0 cM for Stages 1 and 2 and 5.0 cM for Stage 3 (two-dimensional search), excluding positions in the windows of QTL already in the model. In the discussion below, the term *main QTL* refers to a QTL position that has statistically significant main effect. The term *epistatic QTL* refers to a QTL position that has no or small main effect but statistically significant interaction effect with another QTL position.

#### Stage 1: Main QTL and interactions among main QTL

An MIM genome-wide search for QTL with main effects is performed, incorporating the search for interaction among main QTL. Preliminary results showed that incorporating significant interaction parameters in the search improves the power to detect main QTL as well as the power to detect epistasis. After a new QTL is added to the model, the score statistic for each pairwise interaction between the new QTL and the QTL already in the model is computed. The interaction parameter with the maximum score statistic is chosen and the score-statistic re-sampling point-wise threshold is used to test whether to include the interaction parameter in the model. The process is repeated with the new model until there are no more significant interaction parameters that can be added. Backward elimination is then performed on main and interaction parameters. If a QTL is eliminated due to insignificant main effect, any interaction parameters associated with that QTL are also eliminated. To optimize the position of a QTL in the model, score statistics for substitute positions in a 1cM grid of the associated QTL window are computed and the position with the maximum score statistic is selected as the optimized position of the QTL.

#### Stage 2: Epistatic QTL interacting with main QTL

Assume that all significant main QTL, along with significant interactions among those main QTL, have been found. The next stage searches for other QTL whose main effect may be undetectable but which may have significant interaction with one or more other identified QTL. Since QTL with main effects large enough to be detected are already in the model, further contributions to the genetic variance should be due mostly to interaction parameters. Thus the significance test in further searches focuses on interaction parameters.

For each main QTL in the model, positions in the genome grid (avoiding positions that are within QTL windows for QTL in the model) are searched. Consider the full model obtained from adding a main effect parameter corresponding to the position and an interaction parameter corresponding to epistasis between the proposed QTL at the position and the main QTL. The score statistic is computed where the reduced model sets to zero the interaction parameter between the proposed new QTL and the main QTL. The position with the maximum score statistic is identified and the associated p-value for this observed maximum score statistic is computed from the corresponding genome-wide re-sampled distribution. The position and the main QTL corresponding to this minimum p-value is computed. In testing for significance, a Bonferroni adjustment for the number of main QTL in the model is performed. If the adjusted p-value is significant, a new QTL at the position and an interaction term between the new QTL and the main QTL is added to the model.

Once a new QTL and interaction parameter are added to the model, searches are performed for other significant interaction parameters between the new QTL and the previously existing QTL. The procedure is the same as described in Stage 1 for interaction parameters. Including additional interactions before proceeding will avoid falsely detecting a new QTL position that tries to account for an interaction with a QTL already in the model.

The Stage 2 procedure is repeated with the new model until no more significant epistasis can be found. This process however will not find all epistatic QTL since there may be otherwise undetectable QTL pairs that have significant interaction with each other (hence the need for Stage 3).

Before proceeding to Stage 3, the Stage 2 model is optimized. The first step is optimization of the positions of the QTL. To optimize the position of an identified QTL, positions in the corresponding QTL window are scanned. For each substitute position, the score statistic with the reduced model is obtained by setting equal to zero the parameter corresponding to the main effect of the QTL at the position as well as all effect parameters corresponding to the interaction with this QTL. The position with the maximum score statistic is selected to be the optimized position of the QTL. After optimizing positions, backward elimination is performed as described in Stage 1, except that it is performed first on interaction terms and then on main QTL effects for which no interaction effects are left in the model.

#### Stage 3: Interacting QTL with little or no main effect

There may still be undetected epistasis due to possible epistatic QTL pairs, neither of which have a detectable main effect. For this situation a two-dimensional search is performed.

The grid spacing for the two-dimensional search is 5.0 cM, excluding positions in the windows of QTL already in the model. For each position pair in the grid, the main effect parameters for and the interaction parameter between the two proposed QTL are added to the model, considered to be the full model. In computing the score statistic, the reduced model corresponds to setting the interaction parameter equal to zero. The (two-dimensional) genome-wide threshold for the maximum score statistic re-sampled distribution is computed. If the observed maximum score statistic is above the threshold, the two new QTL and corresponding interaction parameter are considered for addition to the model. However, before adding these to the model, the new positions are optimized. This is done by first fixing one of the QTL positions and, using a 1.0 cM grid, finding the best position (based on maximum score statistic) for the second QTL. The position of the second QTL is then fixed and the position of the first QTL is optimized. After admitting a new pair of QTL into the model along with the corresponding interaction parameter, searches for significant interactions between the new QTL and QTL in the model (as described in Stage 2) are performed. The Stage 3 procedure is repeated until no new significant main QTL can be found.

For the final model, an optimization is performed at the end of Stage 3: first optimizing positions of the QTL, then performing backward elimination on interaction effects, and then performing backward elimination on main QTL that have no interaction effect.

### Measures of model fit

We investigate several measures of model fit for the simulation study. Two primary measures, both for QTL and for epistasis, are false positive rate (FPR) and power to detect. These measures require a definition of “misidentified” QTL and a definition of when a simulated QTL is “detected”.

After a final model is determined (for a replicate), each simulated QTL is paired with the nearest QTL on the same chromosome identified in the model. An identified QTL is said to be a *misidentified QTL* if it is not paired with a simulated QTL; otherwise it is said to be a *correctly identified QTL*. A simulated QTL *Q*_*k*_ is said to be a *detected QTL* if it is paired with an identified QTL.

An identified interaction parameter between two identified QTL is said to be a *correctly identified interaction* if each of the identified QTL are correctly identified with a corresponding simulated QTL and the corresponding interaction between the simulated QTL is included in the simulated model; otherwise, it is said to be a *misidentified interaction*. A simulated interaction between simulated QTL *Q*_*k*_ and *Q*_*l*_ is said to be a *detected interaction* if there is a correctly identified QTL paired with *Q*_*k*_ and a correctly identified QTL paired with *Q*_*l*_ and the interaction term between these identified QTL is included in the final model.

It is important to note that our FPR computations are based on individual QTL identification and not on correct model size identification. Our method is not designed to control for a prescribed false positive rate. The FPR measures obtained from the simulations are used to evaluate the performance of the method and to give a practical, empirical sense of expected false positives.

*False Positive Rate for QTL*:

$$\text{FPR} =\frac{\text{\#\ of\ misidentified\ QTL\ over\ all\ replicates}}{\text{total\ \#\ of\ QTL\ identified\ in\ all\ replicates}} $$

*False Positive Rate for Interaction Parameters*:

$$ \text{FPR}_{\mathrm{I}} =\frac{\text{\# of misidentified interactions over replicates}}{\text{total \# of identified interactions over replicates}} $$

*Power to Detect QTL*: For each simulated QTL *Q*_*k*_, compute

$$P(Q_{k}) = \frac{\text{\# of replicates in which}\,\,Q_{k}\,\, \text{is detected}}{\text{total \# of replicates}} $$

*Power to Detect Interaction Parameters*: For each simulated interaction *I*_*k*,*l*_ between *Q*_*k*_ and *Q*_*l*_, compute

$$P(I_{k,l}) = \frac{\text{\# of replicates in which}\,\, I_{k,l} \,\,\text{is detected}}{\text{total \# of replicates}} $$

*Position Estimates*: For each simulated QTL *Q*_*k*_, the use of LOD-support-intervals as confidence intervals for the position estimates is investigated. Manichaikul *et al.* [21] showed that, for interval mapping with one QTL, LOD-1.5 support intervals gave appropriate coverage as 95% confidence intervals. LOD-*z* support intervals for *z*=1,1.5,2 are investigated.

A LOD-*z* support interval around an identified QTL is the longest contiguous interval of positions in which a LOD score is within *z* of the maximum LOD. (The position of the QTL is adjusted to correspond to the maximum LOD score.) The LOD profile for an identified QTL is calculated by comparing the full model with the reduced model where the main effect parameter for the identified QTL and interaction effect parameters with the identified QTL are set to zero. In the case of more than one identified QTL on a chromosome or if the QTL position is near the end of the chromosome, the LOD support interval may have an endpoint determined by the position of a neighboring identified QTL or by the end of a chromosome even if the LOD score has not dropped the specified amount.

For each simulated QTL *Q*_*k*_:

Position estimate for *Q*_*k*_ = average of position estimates for *Q*_*k*_ over replicates for which *Q*_*k*_ is detected.

*LOD-Support-Interval Coverage* = the percentage of replicates for which *Q*_*k*_ is declared detected that *Q*_*k*_ is in the LOD-*z* support interval of the associated identified QTL.

*LOD-Support-Interval Width*: = average over replicates of the width of the LOD-*z* support interval when *Q*_*k*_ is declared detected and *Q*_*k*_ is in the LOD-*z* support interval of the associated identified QTL.

The simulation results provide evidence for whether the LOD-support-interval can be interpreted as a confidence interval. In this case, the width of the LOD-support-interval can be interpreted as a measure of the precision of the position estimate.

*Parameter Estimates*: Given a final model for a replicate, parameter estimates are computed. For a simulated QTL (or interaction), any replicate for which the simulated QTL (or interaction) is not detected is deleted from consideration. For this subset of replicates associated with the simulated QTL (or interaction), we compute the mean of the effect size parameter estimates and the observed standard deviation from this mean.

### Simulations

Simulations were performed for backcross populations. Some initial simulations were done to assess the basic behavior of the use of the score statistic and associated thresholds. These are described in the Results section.

For the simulations, genetic architectures were chosen that were complex enough to highlight the main issues. Architecture 1 was used as a control case to assess the basic performance of the stages. Architecture 2 modified this basic architecture to more closely simulate a practical situation. Detailed information is included in Tables 1 and 2 in the Results section. Both architectures contained 8 QTL on 9 chromosomes where each chromosome was 110 cM with 12 markers placed every 10 cM. Eight interactions among the QTL were simulated. Two of the chromosomes contained two QTL each (to include linkage effects).

**Table 1:** **Observed statistical power of QTL additive and epistatic effect detection and effect estimation from simulation with architecture 1**

**QTL**	**Chr:cM**	**Eff**	**Pw11**	**Pw12**	**Pw21**	**Pw22**	**Pw31**	**Pw32**	**EffE**		**PosE**		**Cov**	**Width**
1	1:47	1.41	100.0	98.3	100.0	100.0	100.0	100.0	1.32	(.02)	44.09	(.60)	100	9.30
2	1:80	1.41	99.0	99.0	100.0	100.0	100.0	100.0	1.47	(.13)	79.96	(.25)	91.6	3.99
3	2:49	0.00	11.3	0.0	87.3	11.7	100.0	100.0	0.24	(.07)	45.24	(.59)	92.6	10.20
4	3:22	1.41	100.0	100.0	100.0	100.0	100.0	100.0	1.41	(.22)	21.99	(.69)	90.0	5.50
5	3:78	0.00	10.3	0.3	88.3	88.3	88.3	88.3	0.02	(.02)	76.93	(.33)	99.3	9.96
6	6:5	0.00	13.3	0.0	11.7	11.7	100.0	100.0	-0.04	(.06)	7.50	(1.1)	92.6	12.74
7	7:70	1.41	100.0	100.0	100.0	100.0	100.0	100.0	1.41	(.03)	69.91	(.31)	100.0	5.07
8	9:63	0.00	12.7	0.0	88.3	88.3	88.3	88.3	0.07	(.04)	63.25	(1.1)	100.0	7.93
FPR	0.124			0.000	0.030	0.024	0.038	0.037						
#QTL	(std	1.18)	5.10	3.99	6.97	6.15	8.08	8.06						
**EPIS**	**Q1:Q2**	**Eff**	**Pw11**	**Pw12**	**Pw21**	**Pw22**	**Pw31**	**Pw32**	**EffE**					
1	1:2	1.77	90.3	89.3	91.7	96.0	96.0	96.3	1.90	(.38)				
2	2:4	1.77	94.7	91.7	92.3	92.3	92.3	92.3	1.68	(.49)				
3	2:7	-1.77	95.7	93.3	93.3	93.3	93.3	93.3	-1.64	(.44)				
4	3:6	1.77	0.0	0.0	11.7	11.7	100.0	100.0	1.79	(.08)				
5	4:5	-1.77	0.7	0.0	88.0	88.0	88.0	88.0	-1.35	(.50)				
6	4:8	1.77	3.0	0.0	88.3	77.7	77.7	77.7	1.42	(.76)				
7	5:7	1.77	1.0	0.0	88.0	88.0	88.0	88.0	1.36	(.51)				
8	7:8	-1.77	3.0	0.0	88.3	88.3	88.3	88.3	-1.74	(.63)				
FPR	0.316			0.001	0.160	0.031	0.053	0.036						

The upper panel shows the results for QTL additive effects and the lower panel shows the results for QTL epistatistic effects. Total heritability (*h* ^2^) is 0.8 with the additive component 0.5 and the epistatic component 0.3. Chr:cM is the QTL chromosome and cM position. Q1:Q2 is the interacting QTL pair. Eff is the simulated effect. Pw11 is the observed statistical power from Stage 1 forward search, Pw12 is that from Stage 1 after optimization and elimination; Pw21 is from Stage 2 forward search, Pw22 is from Stage 2 after the final step of backward elimination of QTL; Pw31 and Pw32 are from the similar steps in Stage 3. EffE is the average effect size estimate and PosE is the average position estimate. (Standard deviations are in parentheses.) Cov is the percent coverage for LOD-1 support interval and Width is the average width in cM of the LOD-1 support interval. FPR is false positive rate. #QTL row records average number of QTL found.

**Table 2:** **Observed statistical power of QTL additive and epistatic effect detection and effect estimation from simulation with architecture 2**

**QTL**	**Chr:cM**	**Eff**	**Pw11**	**Pw12**	**Pw21**	**Pw22**	**Pw31**	**Pw32**	**EffE**		**PosE**		**Cov**	**Width**
1	1:47	1.42	99.3	88.5	88.5	88.5	88.5	100.0	1.27	(.16)	47.1	(1.1)	99.3	8.9
2	1:80	0.76	89.7	89.1	96.0	96.0	96.0	84.5	0.69	(.30)	81.7	(1.1)	100.0	15.3
3	2:49	-0.04	7.1	0.0	0.0	0.0	48.1	48.1	-0.11	(.12)	54.6	(1.8)	97.8	21.6
4	3:22	-0.46	64.9	53.1	54.1	54.1	61.9	61.9	-0.37	(.29)	23.9	(0.7)	100.0	8.4
5	3:78	0.04	6.1	0.0	54.0	54.0	61.8	61.8	0.01	(.04)	78.2	(0.6)	100.0	11.6
6	6:5	-0.06	8.2	0.0	0.0	0.0	47.8	47.8	-0.08	(.08)	2.4	(0.9)	100.0	15.8
7	7:70	0.83	100	100	99.9	99.9	99.9	99.9	0.68	(.04)	69.2	(0.7)	99.0	13.3
8	9:63	0.13	11.1	0.0	54.0	54.0	54.0	54.0	0.01	(.03)	61.5	(2.9)	99.5	16.5
FPR	.08			0.0	0.0	0.0	.004	.004						
#QTL	(std	2.36)	4.20	3.38	4.47	4.47	5.60	5.60						
**EPIS**	**Q1:Q2**	**Eff**	**Pw11**	**Pw12**	**Pw21**	**Pw22**	**Pw31**	**Pw32**	**EffE**					
1	1:2	0.49	15.9	0	0	0	0	0	-	-				
2	2:4	0.99	20.9	0	0	0	0.7	0	-	-				
3	2:7	-0.82	45.8	23.6	23.7	12.9	12.9	8.6	-0.08	(.28)				
4	3:6	-1.63	0	0	0	0	47.8	47.8	-0.59	(.62)				
5	4:5	-1.31	0	0	53.9	53.9	61.7	61.8	-1.17	(.92)				
6	4:8	1.14	0.7	0	53.9	53.9	53.9	54.0	0.79	(.73)				
7	5:7	0.98	0.5	0	48	20.8	23.5	23.5	0.23	(.41)				
8	7:8	-0.98	1.7	0	53.9	53.5	53.5	53.5	-0.52	(.49)				
FPR	.313			.123	.125	.013	.079	.030						

The upper panel shows the results for QTL additive effects and the lower panel shows the results for QTL epistatistic effects. Total heritability (*h* ^2^) is 0.6 with additive component 0.375 and epistatic component 0.225. Chr:cM is the QTL chromosome and cM position. Q1:Q2 is the interacting QTL pair. Eff is the simulated effect. Pw11 is the observed statistical power from Stage 1 forward search, Pw12 is that from Stage 1 after optimization and elimination; Pw21 is from Stage 2 forward search, Pw22 is from Stage 2 after the final step of backward elimination of QTL; Pw31 and Pw32 are from the similar steps in Stage 3. EffE is the average effect size estimate and PosE is the average position estimate. (Standard deviations are in parentheses.) Cov is the percent coverage for LOD-1 support interval and Width is the average width in cM of the LOD-1 support interval. FPR is false positive rate. #QTL row records average number of QTL found.

For Architecture 1, overall heritability in the broad sense was *h*^2^=0.8 with additive component 0.5 and epistatic component 0.3. There were four QTL with detectable main effect (equal effect sizes) and the eight interaction effect sizes were equal. Two other QTL were assigned zero main effect but had interaction with main QTL. Two other QTL were assigned zero main effect with interaction with each other and not with any other QTL. For Architecture 2, the simulated positions of the QTL and the chosen interactions were the same as in Architecture 1. To more closely simulate a practical situation, the heritability was lowered to *h*^2^=0.6 (with additive component 0.375 and epistatic component 0.225). In addition, the effect sizes (main as well as interaction) were chosen to be variable, maintaining the structure of four main QTL with four much smaller effect size QTL, two of which interacted with the some of the main QTL, and two of which interacted only with each other with large interaction effect (same positions as in Architecture 1).

For each simulation the sample size was 300 individuals. For Architecture 1, 300 replicates were used and, for Architecture 2, 1000 replicates were used. For each replicate, 1000 resampling steps were used in computing score statistic thresholds. The trait values were generated with a residual error following the standard normal distribution *N*(0,1).

### Instruction for using the procedures in Windows QTL Cartographer

A completely new Multiple Interval Mapping (MIM) method has been implemented in Windows QTL Cartographer (http://statgen.ncsu.edu/qtlcart/WQTLCart.htm). This new MIM method uses the search strategy and statistical test developed from this study. The method has a number of procedures that perform different functions and can be used interactively for practical data analysis. These procedures include the following components.

1. MIM forward search procedure: This is an automatic QTL search procedure that is intended for generating an initial MIM model for further analysis only. QTL is searched sequentially based on its main effect and added into the model subject to a score-statistic test with a genome-wise threshold. Upon detecting a new QTL, interaction effects of the new QTL with the previously identified QTL are tested and added into the model using a score-statistic test with a point-wise threshold.
2. Optimizing QTL positions with or without interaction effects: This procedure optimizes position estimate of each QTL in turn. When the option with interaction effects is chosen, both the main effect of the QTL and interaction effects with other QTL are used for optimizing the estimate of position. Otherwise, only the main effect of the QTL is used for optimizing the QTL position.
3. Search for New QTL:
  - QTL with main effects: This procedure searches for a new QTL based on a score-statistic test on the main effect with a genome-wise threshold.
  - QTL with interaction effects:
    i. Search for interaction effects among identified QTL: This procedure searches for significant interaction effects among identified QTL using a point-wise threshold.
    ii. Search for new QTL that have significant interaction effects with identified QTL: This procedure searches for a new QTL based on its interaction effect with an identified QTL. A one-dimensional genome scan is performed to search for the best position for a new QTL that has an interaction effect with any identified QTL. This interaction effect is tested by a score-statistic test subject to a threshold that takes into account the search space (genome-wide with multiple identified QTL).
    iii. Search for new QTL in pair that have significant interaction effects: This procedure performs a two-dimensional genome scan that searches for the best positions for a pair of new QTL that have significant interaction effect(s). The interaction effect(s) are tested based on a threshold that takes into account the search space (the two-dimensional genome-scan).
4. Testing QTL effects:
  - Testing QTL main effects: This procedure tests for significance the main effects of QTL in the current MIM model. If a QTL main effect is not significant at a genome-wise significant level, the QTL will be eliminated from the model. The procedure can be used only for those QTL that do not have interaction effects with other QTL to avoid eliminating QTL that have weak main effects but significant interaction effects with other QTL.
  - Testing QTL interaction effects: This procedure tests for significance the interaction effects of QTL in the current MIM model. If an interaction effect is not significant at a point-wise significant level, the interaction effect will be eliminated from the model.
5. Estimating QTL effects: This procedure produces test statistics (log-likelihood ratio test statistic and score statistic) and empirical p-value of the score statistic for each QTL effect in the current MIM model.
6. Producing summary output: This procedure produces a comprehensive report of information of the current MIM model in two output files. One output file includes estimates of QTL number, positions, main and interaction effects, *R*^2^ value of the model (an estimate of the broad-sense heritability) and partition of the *R*^2^ value into the variance components due to individual QTL main and interaction effects and the covariance components due to a pair of QTL effects (due to linkage disequilibrium), Equation (19) of [20]. It also includes estimates of QTL genotypes and genotypic values of the trait for each individual, Equation (14) of [20]. The other output file includes information for generating the log-likelihood profile of each QTL in the MIM model in a graphic which displays automatically. The log-likelihood profile for each QTL utilizes the combined information of the main effect of that QTL and interaction effects of the QTL with other QTL in the model.

For practical data analysis, we recommend use these procedures in the following way.

- Procedure 1 can be used to search for an initial model.
- Procedure 2 can be used next to optimize QTL position estimates. Procedure 2 can be used repeatedly when the model structure is changed by adding or removing a QTL during the model fitting process.
- Before searching for new QTL, current QTL effects should be checked by first using procedure 4(b), then 4(a).
- Procedure 3(a) can be used to search for new QTL based on main effects. This procedure can be used for multiple times in conjunction with procedure 2 until no new QTL based on main effects can be found.
- Procedure 3(b)(1) can be used to search for significant interaction effects among identified QTL.
- Then procedure 3(b)(2) can be used to search for additional QTL that have significant interaction effects with the other identified QTL. If an additional QTL is identified, procedure 2, 4(b) and 4(a) can be used to optimize the model and check the model again.
- Only after other procedures have been used repeatedly, i.e. QTL that have significant main effects or have significant interaction effects with the main effect QTL have already been identified and fitted in the model, should procedure 3(b)(3) be used to search for additional new QTL that have significant interaction effects only. Procedure 3(b)(3) should be used only in the last stage to minimize the risk of mapping epistatic QTL in wrong positions due to other unaccounted linked QTL effects. This point cannot be overemphasized enough.
- Procedure 6 can be used to generate a report for a MIM model.

## Results

The model selection is in three stages: (1) search for main QTL along with interaction effects among the main QTL, (2) search for epistatic QTL with significant interaction with a main QTL, and (3) two-dimensional search for other epistatic QTL pairs (where neither QTL has significant main effect). At the end of each stage, we optimize the model using backward elimination and optimization of position. Without optimization, succeeding stages will tend to identify extraneous QTL or interactions that would be difficult to eliminate later. The completion of each stage then minimizes the residual variance and potential bias, thus increasing the power of detection for the next stage.

Tables 1 and 2 describe the simulated architectures (Architecture 1 and Architecture 2 respectively) and display the final power, FPR and estimate results as well as corresponding intermediate results of power at each stage of the model selection. The top half of the table describes the architecture and results for QTL additive effects. The bottom half of the table describes the architecture and results in a similar manner for interaction effects between QTL Q1 and Q2.

The overall stability of the model selection process can be assessed from the sequence of FPR and power (Pw) for both architectures. The backward elimination and optimization stages are critical. The Stage 1 forward search for QTL and epistasis generated a high FPR (for both main QTL and epistatic parameters) (column Pw11) which was considerably reduced by the elimination/optimization phase with minimal impact on power (Pw12 column). This indicates that the standard two-dimensional forward search strategy would almost certainly perform poorly. Equally important to note is that further genome-wide searches (Stages 2 and 3) to account for epistasis did not introduce substantial increases in FPR even though there were many chances to detect false interactions. These results supported the model selection philosophy of proceeding in stages.

The overall effectiveness of the model selection process can be seen in several ways. Stage 2 detected QTL that have little or no main effect but interact with main QTL (QTL 5 and 8) and Stage 3 found QTL pairs that have strong interaction but little or no main effects (QTL 3 and 6), as they were designed to do. An equally important observation is that the power to detect QTL was increased by the detection of more epistasis. This can be seen most clearly in the increase in power from Stage 2 to Stage 3 for QTL 4 and 5 in Architecture 2. This is also the case for the increase of detecting QTL 2 at Stage 2 due to the interaction between QTL 2 and 7 in Architecture 2.

The final FPR was well within the nominal significance level of 0.05. Model selection was not designed to control FPR; however, the FPR results were meant to give a practical, empirical sense of expected false positives. The results also suggested that a larger *α* significance level could be used to increase power while still maintaining an acceptable level of FPR. We compared final FPR and power results for Architecture 2 using *α*=0.2 with previous results using *α*=0.05, and FPR is still maintained below 0.05 while the dection power is improved (results not shown).

Model size was another measure of stability of the procedure. For Architecture 1, the average number of QTL found at each stage of the process was as expected – for stage 1, 4 QTL with main effect were detected; for stage 2, 6 QTL with main effect or interacting with main QTL were detected; for stage 3, the number of simulated QTL (8) were detected (see #QTL row in Table 1). Additional evidence was that 77% of the 300 replicates identified 8 QTL and 84% of the replicates identified 8 interaction terms (the number of simulated interaction terms).

Another indication of effectiveness of the method was the assessment of confidence in position estimates. There is evidence in the literature that LOD support intervals can function as confidence intervals [21]. Coverage is the percentage of the replicates for which a simulated QTL is declared detected. The last two columns (Cov and Width) in Tables 1 and 2 record the LOD-1 interval coverage and width in cM respectively. The high coverage provided strong evidence that the LOD-support-interval can be interpreted as a confidence interval and that the width of the LOD-support-interval can be interpreted as a measure of the precision of the position estimate.

Results also gave some insight into effects of linkage and multi-interactions. For example, in Architecture 1 results (where simulated effects were equal), QTL 5 and 8 were detected due to interactions as are QTL 3 and 6, since they have zero main effect. The power to detect QTL 3 and 6 was very high whereas the power to detect QTL 5 and 8 was lower. For QTL 3 and 6, there was only one interaction (3,6). On the other hand, QTL 5 and 8 each interact with two other QTL (4 and 7) so the effects were harder to disentangle. Another indication of the effect of multi-interactions was the somewhat lower LOD coverage for QTL 4 (Architecture 1) as QTL 4 interacts with three other QTL.

**Figure 1:**
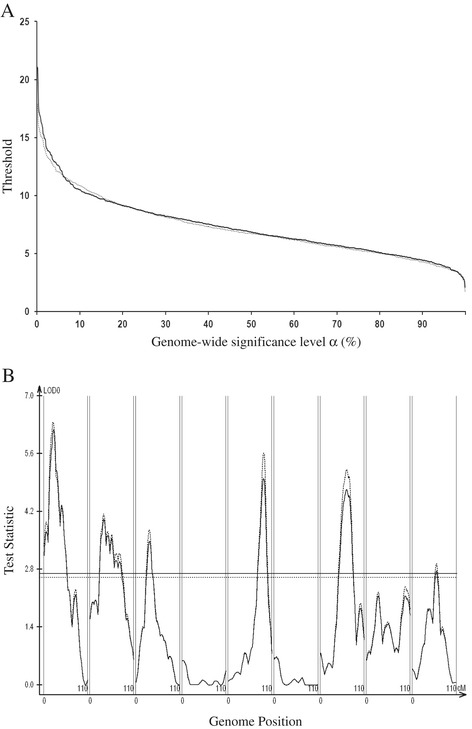
**Comparison of score statistic and threshold with likelihood ratio statistic and permutation threshold.** **A**, compares threshold values (y-axis) across significance levels *α* (x-axis) with score threshold indicated by the dotted curve and permutation threshold indicated by the solid curve. **B**, compares the likelihood ratio profile (solid) and score statistic profile (dotted) for one replication; the solid and dotted horizontal lines represent the permutation threshold and score threshold (*α* = 5%), respectively.

**Figure 2:**
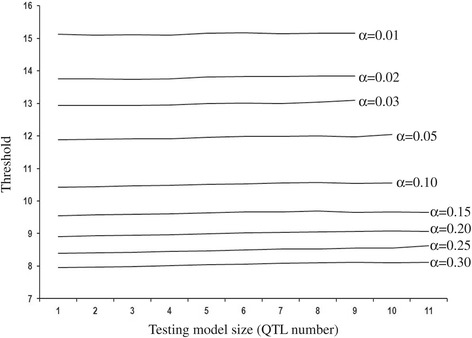
**Score threshold as QTL are added to the model (averaged over replicates).** The x-axis represents the number of QTL in the model being tested. Each line represents a significance level *α*: from top to bottom, *α* = 0.01, 0.02, 0.03, 0.05, 0.10, 0.15, 0.20, 0.25, 0.30.

Interval mapping for detecting one QTL using the likelihood ratio statistic and permutation threshold is well established in the literature [15]. In a sperate simulation of the null model of no QTL, the score procedure for selecting the first QTL behaved as expected with respect to Type 1 error, as that for the permutation test (Figure 1), reinforcing the comparison results of [15]. Of couse, in this study we extended the score statistic for all the three-stages and multiple steps within the statges. It turns out that score thresholds are very similar at different steps of search process for QTL as shwon in Figure 2 for stage 1, demonstaing that the score statistic genome-wide threshold mainly depends on genome size and not on model size (QTL number).

## Discussion

Mapping QTL with epistatic effects efficiently and reliably has been a persistent problem for QTL mapping analysis. There are a number of problems for studying epistatic QTL. Sample size for many QTL mapping experiments is generally relatively small for an ideal study of QTL epistasis. Linkage can impose a significant challenge for finding epistatic QTL reliably. If multiple QTL are in linkage and have interactions, searching for QTL can become a very delicate issue.

One strategy for analyzing QTL epistasis is to perform a two-dimensional genome scan for a pair of QTL with epistasis [1,3]. There are two potential problems for this strategy. If there are multiple QTL in linkage and with complex interaction patterns, a two-dimensional search can yield some apparent “epistatic QTL” at wrong positions. Also the statistical power for the search is likely to be low as the genetic variance due to other unmapped QTL is in the residual and not fitted in the model.

To tackle the problem of complex interaction of multiple QTL with linkage, we experimented with a number of search strategies (unpublished) and finally settled on a three-stage search strategy. In the first stage, the main effect QTL are searched and mapped. The significant epistatic effects of identified QTL are also estimated. In the second stage, epistatic QTL that interact significantly with other identified QTL are searched. In the third stage, new epistatic QTL are searched in pairs.

There are a number of justifications for this search strategy. First by the principle of least squares in the partition of variance, the most genetic variance is due to the marginal or main effects of QTL. Thus by first mapping those QTL, the statistical power for the second and third stages of analysis for mapping epistatic QTL can be maximized. Also, by the property of the orthogonal genetic model, the main and interaction effects of QTL are uncorrelated even with linkage in the backcross and recombinant inbred populations [11]. This means that epistasis does not necessarily bias the search of main effect QTL in the first stage of analysis. This is clearly observed in our simulation study. Statistically the main effect QTL are relatively easier to be identified and can be effectively searched through a stepwise procedure. After exhaustively searching for main effect QTL, the task of searching for epistatic QTL can become relatively easier, more powerful and robust. Again, we first take care of those epistatic QTL that interact with the main effect QTL, because they are relatively easier to be identified. Only after all other QTL have been exhaustively searched, should an attempt be made at the last stage to search for QTL that do not have significant main effects, but significant interaction effects. Failure to exercise this precaution can lead to the identification of spurious epistatic QTL that could be due to other unmapped QTL effects.

One observation made in our simulation study is the critical importance of performing backward elimination of non-significant spurious QTL at the end of each stage before proceeding to the next stage. Without this procedure, false positive QTL detection can be significantly increased.

A major component of the new method is the use of a score-statistic re-sampling procedure for empirically estimating thresholds for model selection [15]. This procedure is data-based, model-based, computationally efficient, and flexible for different search procedures. It takes into account the data structure and the search space at different search stages for different combinations of parameters. Thus it provides dynamic and relevant criteria for model selection during the search process.

## Conclusions

We have developed a new QTL mapping method that can effectively map multiple QTL with complex linkage and interaction patterns. The simulation study demonstrated the capability of the new method in identifying complex epistatic QTL reliably and powerfully while keeping false positive identification at a low level. The method has been implemented in Windows QTL Cartographer V2.5 that is freely distributed for general QTL mapping data analysis [16]. The method as implemented has a number of procedures that perform different functions and can be used interactively in practical data analysis. We include the instruction on how to use the procedures in the Methods section.

## Additional file

Additional file 1
**Supplementary Methods [** 15 **,** 19 **].**

## Abbreviations

QTL: Quantitative trait loci
LOD: Log of odds
BIC: Bayesian informatiion criterion
EM: Expetation and maximization
MIM: Multiple interval mapping
FPR: False positive rate
