# Supplementary material for: Mapping epistatic quantitative trait loci
Source: BMC Genet. 2014 Nov 4;15:112. doi: 10.1186/s12863-014-0112-9 (PMC4226885; doi:10.1186/s12863-014-0112-9)
Supplement: Additional file 1 — Supplementary Methods [ 15 , 19 ]. [file 12863_2014_112_MOESM1_ESM.pdf]

Additional File 1: Supplementary Methods for "Mapping Epistatic Quantitative Trait Loci"

Cecelia Laurie[1], Shengchu Wang[2], Luciana Aparecida Carlini-Garcia[3], Zhao-Bang Zeng[2,4,*]

1 Department of Mathematics, University of Alabama, Tuscaloosa, AL, USA and Department of Biostatistics, University of Washington, Seattle, WA, USA
2 Department of Statistics & Bioinformatics Research Center,North Carolina State University, Raleigh, North Carolina, USA
3 Instituto Agronômico de Campinas, Centro de Grãos e Fibras, Campinas, SP, Brazil and APTA Regional, Pólo Centro Sul, Piracicaba, SP, Brazil
4 Department of Statistics & Bioinformatics Research Center and Department of Genetics, North Carolina State University, Raleigh, USA
∗ E-mail: zeng@stat.ncsu.edu

Contents

1 Supplementary Methods **1**
1.1 Model 1
1.2 Score statistic and re-sampling 2
1.3 Partial derivatives 4

1 Supplementary Methods

1.1 Model

For the backcross model as described in the paper, assume that there are m QTL in the model with t epistatic effects for a total of $c = m + t$ effects parameters. The likelihood is a mixture of 2^m normal distributions, one for each of the possible missing QTL genotypes.

The individual and the overall log-likelihoods are given by

$$l_i(\theta; \mathbf{v}) = ln \left\{ \sum_{j=1}^{2^m} p_{ij} \phi(y_i | \mu_j, \sigma^2) \right\}$$

$$l(\theta; \mathbf{v}) = \sum_{i=1}^{n} ln \left\{ \sum_{j=1}^{2^m} p_{ij} \phi(y_i | \mu_j, \sigma^2) \right\}$$

We have that $\mathbf{v}$ denotes the vector of positions of the QTL and θ denotes the vector of main and epistatic effects parameters along with the overall mean μ and the variance

σ^2 of the residual effects. Recall that $\phi(y_i|\mu_j, \sigma^2)$ denotes the normal distribution with mean μ_j and variance σ^2 and that the mixing proportion p_{ij} is the probability of the jth multilocus genotype conditioned on marker data.

Genetic Design Matrix D:
Let $\mathbf{D}= [d_{jp}]_{2^m \times c}$ where row j corresponds to the jth multilocus QTL genotype and column p corresponds to the pth effects parameter. For $p \leq m$, $d_{jp} = \frac{1}{2}$ or $-\frac{1}{2}$ depending upon whether the pth locus in the multilocus QTL genotype j is homozygous or, respectively, heterozygous. For $p > m$, $d_{jp} = \frac{1}{4}$ if the two interacting QTL in the multilocus genotype j are both homozygous or both heterozygous and $d_{jp} = -\frac{1}{4}$ if one of the QTL is homozygous and the other heterozygous.

Effects Matrix E:
Let $\mathbf{E}$ be the $c \times 1$ vector of the effects parameters, i.e. $\mathbf{E} = [a_1; \ldots; a_m; \gamma_1; \ldots; \gamma_t]$ where $a_1, \ldots, a_m$ are the main effects parameters and $\gamma_1, \ldots, \gamma_t$ are the epistasis effects parameters.

Genotypic Value μ_j:
Assuming multilocus genotype j, we have that $\mu_j = \mu + \mathbf{D}_j\mathbf{E}$ where $\mathbf{D}_j$ denotes the jth row of $\mathbf{D}$

Mixing Probabilities Matrix $\mathbf{\Pi}$:
Let π_{ij} denote the probability of multilocus QTL genotype j conditional on marker genotypes and also phenotypic value, for individual i. We have that

$$\pi_{ij} = \frac{p_{ij}\phi(y_i|\mu_j, \sigma^2)}{\sum_{j=1}^{2^m} p_{ij}\phi(y_i|\mu_j, \sigma^2)}$$

Let $\mathbf{\Pi}$ be the $n \times 2^m$ matrix whose (i, j) entry is π_{ij}.

For further derivation and discussion of the above quantities, see [1].

1.2 Score statistic and re-sampling

We describe the score statistic and the re-sampling procedure. We denote by l_i the log-likelihood of individual i. Let D be a set of indices representing the set of models examined. For a given model (represented by $d \in D$), assume that the model has c parameters, split into two groups, $\theta = (\eta, \beta) = (\theta_1, \cdots, \theta_{c-1}, \beta, \mu, \sigma^2)$, where β is the parameter (or parameters) to be tested for significance and $\eta = (\theta_1, \cdots, \theta_{c-1}, \mu, \sigma^2)$ is considered the vector of nuisance parameters. In the following, $\widetilde{\eta}$ denotes the maximum likelihood estimator of η for the reduced model with $\beta = 0$.

Score Statistic

$$\widehat{U}_i(d) = U_{\beta,i}(0,\widetilde{\eta};d) - \left(\frac{\partial^2 l_i(0,\widetilde{\eta};d)}{\partial\beta\partial\eta}\right)\left(\frac{\partial^2 l_i(0,\widetilde{\eta};d)}{\partial\eta^2}\right)^{-1} U_{\eta,i}(0,\widetilde{\eta};d)$$

$$U_{\beta,i}(\beta,\eta;d) = \frac{\partial l_i(\beta,\eta:d)}{\partial\beta}$$

$$U_{\eta,i}(\beta,\eta;d) = \frac{\partial l_i(\beta,\eta;d)}{\partial\eta} = (\frac{\partial l_i}{\partial\theta_1},\cdots,\frac{\partial l_i}{\partial\theta_{m-1}},\frac{\partial l_i}{\partial\mu},\frac{\partial l_i}{\sigma^2})'$$

$$\widehat{U}(d) = \sum_{i=1}^{n}\widehat{U}_i(d)$$

The score statistic for H_0: $\beta = 0$ against H_1: $\beta \neq 0$ for model d is

$$W(d) = \widehat{U}'(d)\widehat{V}^{-1}(d)\widehat{U}(d)$$

where $\widehat{V}(d) = \sum_{i=1}^{n}\widehat{U}_i(d)\widehat{U}_i'(d)$. (Here the notation M' refers to the transpose of the matrix M.) Formulas for the needed first and second partial derivatives are given in the subsection *Partial derivatives* below.

Resampling Thresholds

The stages involve forward searches for positions of QTL to add to the model, testing for admitting interaction parameters, and backward elimination phases. The forward searches for positions along the genome require a genome-wide threshold for an empirical distribution of maximum score statistics. Testing for admitting or deleting additional interaction parameters given that QTL are identified or deleting QTL in the model do not involve a genome search and thus we use a pointwise threshold for testing of significance.

To find a ***genome-wide threshold*** using resampled score statistics (when searching for positions along the genome):

1. Find independent samples G_i, $i = 1, 2, \cdots, n$ from $N(0,1)$. Here n is the number of individuals in the sample.

2. Calculate $U^*(d) = \sum_{i=1}^{n}\widehat{U}_i(d)G_i$, $W^*(d) = U^{*'}(d)\widehat{V}^{-1}U^*(d)$, and $S^* = max_d W^*(d)$.

3. Repeat step 1 (choosing a new sample $\{G_i\}$) and step 2 a large number of times, say N times. Let ${S_k}^*$ for $k = 1, \cdots, N$ denote the kth maximum resampled score statistic.

4. Compute the $100(1-\alpha)^{th}$ percentile of $\{{S_k}^* : k = 1, \cdots, \mathrm{N}\}$ to determine the threshold value. Here α is the desired significance level.

Note that the $\widehat{U}_i(d)$ and $\widehat{V}(d)$ used in the resampling calculations are based on the original data and are evaluated once and used repeatedly in step 2; only the G_i's are changed in each resample. Since it does not involve refitting the model in each iteration, the proposed method is computationally much more efficient than the permutation method. More complete explanations and justifications of the procedure can be found in [2].

To find a ***point-wise threshold*** for a given model $d \in D$ (when testing epistasis parameters or parameters in backward-elimination) :

1. Find independent samples G_i, $i = 1, 2, \cdots, n$ from $N(0, 1)$. Here n is the number of individuals in the sample.
2. Calculate $U^*(d) = \sum_{i=1}^{n} \widehat{U}_i(d) G_i$ and $W^*(d) = U^{*'}(d)\widehat{V}^{-1}U^*(d)$.
3. Repeat step 1 (choosing a new sample $\{G_i\}$) and step 2 a large number of times, say N times. Let ${W_k}^*$ for $k = 1, \cdots, N$ denote the kth resampled score statistic.
4. Compute the $100(1-\alpha)^{th}$ percentile of $\{{W_k}^* : k = 1, \cdots, \mathrm{N}\}$ to determine the threshold value. Here α is the desired significance level.

1.3 Partial derivatives

Let $\mathbf{T}$ be the $n \times 2^m$ matrix such that $t_{ij} = y_i - \mu_j$.
Let $\mathbf{S}$ be the $n \times 2^m$ matrix such that $s_{ij} = \dfrac{t_{ij}^2}{2\sigma^2} - \dfrac{1}{2}$.

Throughout what follows, the matrix operator # denotes the Hadamard product of two matrices, i.e. element-wise multiplication. For a matrix G, G_i refers to the ith row of G, $G^{[q]}$ refers to the qth column of G, and G' refers to the transpose of G.

First and second partial derivatives of the log-likelihood functions

Let $\theta = (\theta_1, \ldots, \theta_c, \mu, \sigma^2)$ denote the vector of parameters for the model and let $\mathbf{v}$ denote the vector of positions of the QTL. In what follows we will shorten the notation $l(\theta; \mathbf{v})$ and $l_i(\theta; \mathbf{v})$ to $l(\theta)$ and $l_i(\theta)$ respectively, since it is understood that the likelihood depends on the positions but our focus is on derivatives with respect to the parameters in θ.

Basic building blocks: Assume that $k = 1, \cdots, c$.

$$\frac{\partial(y_i - \mu_j)}{\partial\theta_k} = -d_{jk}$$

$$\frac{\partial(y_i - \mu_j)}{\partial\mu} = -1$$

$$\frac{\partial}{\partial\theta_k}\phi(y_i|\mu_j,\sigma^2) = \frac{\partial}{\partial\theta_k}\left[\frac{1}{\sqrt{2\pi\sigma^2}}e^{-\frac{1}{2}\frac{(y_i-\mu_j)^2}{\sigma^2}}\right] = \phi(y_i|\mu_j,\sigma^2)\frac{1}{\sigma^2}(y_i-\mu_j)d_{jk}$$

$$\frac{\partial}{\partial\mu}\phi(y_i|\mu_j,\sigma^2) = \frac{\partial}{\partial\mu}\left[\frac{1}{\sqrt{2\pi\sigma^2}}e^{-\frac{1}{2}\frac{(y_i-\mu_j)^2}{\sigma^2}}\right] = \phi(y_i|\mu_j,\sigma^2)\frac{1}{\sigma^2}(y_i-\mu_j)$$

$$\frac{\partial}{\partial\sigma^2}\phi(y_i|\mu_j,\sigma^2) = \frac{\partial}{\partial\sigma^2}\left[\frac{1}{\sqrt{2\pi\sigma^2}}e^{-\frac{1}{2}\frac{(y_i-\mu_j)^2}{\sigma^2}}\right] = \frac{1}{\sigma^2}\phi(y_i|\mu_j,\sigma^2)\left(\frac{(y_i-\mu_j)^2}{2\sigma^2}-\frac{1}{2}\right)$$

First partial derivatives of individual log-likelihoods

$$\frac{\partial l_i(\theta)}{\partial\theta_k} = \frac{\partial}{\partial\theta_k}\left[ln\left\{\sum_{j=1}^{2^m}p_{ij}\phi(y_i|\mu_j,\sigma^2)\right\}\right] = \sum_{j=1}^{2^m}\pi_{ij}\frac{1}{\sigma^2}(y_i-\mu_j)d_{jk}$$

$$\frac{\partial l_i(\theta)}{\partial\mu} = \frac{\partial}{\partial\mu}\left[ln\left\{\sum_{j=1}^{2^m}p_{ij}\phi(y_i|\mu_j,\sigma^2)\right\}\right] = \sum_{j=1}^{2^m}\pi_{ij}\frac{1}{\sigma^2}(y_i-\mu_j)$$

$$\frac{\partial l_i(\theta)}{\partial\sigma^2} = \frac{\partial}{\partial\sigma^2}\left[ln\left\{\sum_{j=1}^{2^m}p_{ij}\phi(y_i|\mu_j,\sigma^2)\right\}\right] = \sum_{j=1}^{2^m}\pi_{ij}\frac{1}{\sigma^2}\left(\frac{(y_i-\mu_j)^2}{2\sigma^2}-\frac{1}{2}\right)$$

Second partial derivatives of the full log-likelihood:

We will need the partial derivatives of π_{ij}. In the expressions below we will use the notation $t_{ij} = y_i - \mu_j$ and $s_{ij} = \frac{(y_i-\mu_j)^2}{2\sigma^2} - \frac{1}{2}$.

$$\frac{\partial\pi_{ij}}{\partial\theta_k} = \frac{1}{\sigma^2}\pi_{ij}\left(t_{ij}d_{jk} - \sum_{J=1}^{2^m}\pi_{iJ}t_{iJ}d_{Jk}\right)$$

$$\frac{\partial\pi_{ij}}{\partial\mu} = \frac{1}{\sigma^2}\pi_{ij}\left(t_{ij} - \sum_{J=1}^{2^m}\pi_{iJ}t_{iJ}\right)$$

$$\frac{\partial\pi_{ij}}{\partial\sigma^2} = \frac{1}{\sigma^2}\pi_{ij}\left(s_{ij} - \sum_{J=1}^{2^m}\pi_{iJ}s_{iJ}\right)$$

The following refer to $k = 1, \cdots, c$ and $p = 1, \cdots, c$.

$$\textbf{(1)}\frac{\partial^2 l(\theta)}{\partial\theta_p\partial\theta_k} = \frac{\partial}{\partial\theta_p}\left[\sum_{i=1}^{n}\sum_{j=1}^{2^m}\frac{1}{\sigma^2}\pi_{ij}(y_i-\mu_j)d_{jk}\right]$$
$$= -\sum_{i=1}^{n}\sum_{j=1}^{2^m}\frac{1}{\sigma^2}\pi_{ij}d_{jk}d_{jp} + \sum_{i=1}^{n}\sum_{j=1}^{2^m}\frac{1}{\sigma^2}\frac{\partial\pi_{ij}}{\partial\theta_p}t_{ij}d_{jk}$$
$$= -\frac{1}{\sigma^2}\sum_{i=1}^{n}\sum_{j=1}^{2^m}\pi_{ij}d_{jk}d_{jp} + \left(\frac{1}{\sigma^2}\right)^2\sum_{i=1}^{n}\sum_{j=1}^{2^m}\pi_{ij}t_{ij}^2 d_{jk}d_{jp}$$
$$- \left(\frac{1}{\sigma^2}\right)^2\sum_{i=1}^{n}\left[\left(\sum_{j=1}^{2^m}\pi_{ij}t_{ij}d_{jk}\right)\left(\sum_{J=1}^{2^m}\pi_{iJ}t_{iJ}d_{Jp}\right)\right]$$

In matrix notation, we have:

$$\frac{\partial^2 l(\theta)}{\partial\theta_p\partial\theta_k} = -\frac{1}{\sigma^2}\mathbf{1}_{1\times n}\mathbf{\Pi}(\mathbf{D}^{[k]}\#\mathbf{D}^{[p]}) + \left(\frac{1}{\sigma^2}\right)^2\mathbf{1}_{1\times n}(\mathbf{\Pi}\#\mathbf{T}\#\mathbf{T})(\mathbf{D}^{[k]}\#\mathbf{D}^{[p]})$$
$$- \left(\frac{1}{\sigma^2}\right)^2\mathbf{1}_{1\times n}\left\{[(\mathbf{\Pi}\#\mathbf{T})\mathbf{D}^{[k]}]\#[(\mathbf{\Pi}\#\mathbf{T})\mathbf{D}^{[p]}]\right\}$$

where $\mathbf{1}_{1\times n}$ is the $1 \times n$ matrix whose entries are all equal to 1.

(2) $\frac{\partial^2 l(\theta)}{\partial\mu\partial\theta_k}$ is composed of expressions similar to those in **(1)** except that the d_{jp} (and d_{Jp}) factors are replaced by 1.

In matrix notation, we have:

$$\frac{\partial^2 l(\theta)}{\partial\mu\partial\theta_k} = -\frac{1}{\sigma^2}\mathbf{1}_{1\times n}\mathbf{\Pi}\mathbf{D}^{[k]} + \left(\frac{1}{\sigma^2}\right)^2\mathbf{1}_{1\times n}(\mathbf{\Pi}\#\mathbf{T}\#\mathbf{T})\mathbf{D}^{[k]}$$
$$- \left(\frac{1}{\sigma^2}\right)^2\mathbf{1}_{1\times n}\left\{[(\mathbf{\Pi}\#\mathbf{T})\mathbf{D}^{[k]}]\#[(\mathbf{\Pi}\#\mathbf{T})\mathbf{1}_{2^m\times 1}]\right\}$$

where $\mathbf{1}_{2^m\times 1}$ is the $2^m \times 1$ matrix whose entries are all equal to 1.

(3) $\frac{\partial^2 l(\theta)}{\partial\mu\partial\mu}$ is composed of expressions similar to those in **(2)** except that the d_{jk} (and d_{Jk}) factors are replaced by 1.

In matrix notation, we have:

$$\frac{\partial^2 l(\theta)}{\partial\mu\partial\mu} = -\frac{1}{\sigma^2}\mathbf{1}_{1\times n}\mathbf{\Pi}\mathbf{1}_{2^m\times 1} + \left(\frac{1}{\sigma^2}\right)^2 \mathbf{1}_{1\times n}(\mathbf{\Pi}\#\mathbf{T}\#\mathbf{T})\mathbf{1}_{2^m\times 1} - \left(\frac{1}{\sigma^2}\right)^2 \mathbf{1}_{1\times n}\left\{[(\mathbf{\Pi}\#\mathbf{T})\mathbf{1}_{2^m\times 1}]\#[(\mathbf{\Pi}\#\mathbf{T})\mathbf{1}_{2^m\times 1}]\right\}$$

Note that $\mathbf{1}_{1\times n}\mathbf{\Pi}\mathbf{1}_{2^m\times 1}$ equals n, the sample size.

(4) $$\frac{\partial^2 l(\theta)}{\partial\theta_k\partial\sigma^2} = \frac{\partial}{\partial\theta_k}\left[\sum_{i=1}^{n}\sum_{j=1}^{2^m}\frac{1}{\sigma^2}\pi_{ij}\left[\frac{(y_i-\mu_j)^2}{2\sigma^2}-\frac{1}{2}\right]\right]$$
$$= -\sum_{i=1}^{n}\sum_{j=1}^{2^m}\left(\frac{1}{\sigma^2}\right)^2\pi_{ij}t_{ij}d_{jk} + \sum_{i=1}^{n}\sum_{j=1}^{2^m}\frac{1}{\sigma^2}\frac{\partial\pi_{ij}}{\partial\theta_k}s_{ij}$$
$$= -\left(\frac{1}{\sigma^2}\right)^2\sum_{i=1}^{n}\sum_{j=1}^{2^m}\pi_{ij}t_{ij}d_{jk} + \left(\frac{1}{\sigma^2}\right)^2\sum_{i=1}^{n}\sum_{j=1}^{2^m}\pi_{ij}s_{ij}t_{ij}d_{jk} - \left(\frac{1}{\sigma^2}\right)^2\sum_{i=1}^{n}\left[\left(\sum_{j=1}^{2^m}\pi_{ij}s_{ij}\right)\left(\sum_{J=1}^{2^m}\pi_{iJ}t_{iJ}d_{Jk}\right)\right]$$

In matrix notation, we have:

$$\frac{\partial^2 l(\theta)}{\partial\theta_k\partial\sigma^2} = -\left(\frac{1}{\sigma^2}\right)^2 \mathbf{1}_{1\times n}(\mathbf{\Pi}\#\mathbf{T})\mathbf{D}^{[k]} + \left(\frac{1}{\sigma^2}\right)^2 \mathbf{1}_{1\times n}(\mathbf{\Pi}\#\mathbf{S}\#\mathbf{T})\mathbf{D}^{[k]} - \left(\frac{1}{\sigma^2}\right)^2 \mathbf{1}_{1\times n}\left\{[(\mathbf{\Pi}\#\mathbf{T})\mathbf{D}^{[k]}]\#[(\mathbf{\Pi}\#\mathbf{S})\mathbf{1}_{2^m\times 1}]\right\}$$

(5) $\frac{\partial^2 l(\theta)}{\partial\mu\partial\sigma^2}$ consists of expressions similar to those in **(4)** except that the d_{jk} (and d_{Jk}) factors are replaced by 1.

In matrix notation, we have:

$$\frac{\partial^2 l(\theta)}{\partial\mu\partial\sigma^2} = -\left(\frac{1}{\sigma^2}\right)^2 \mathbf{1}_{1\times n}(\mathbf{\Pi}\#\mathbf{T})\mathbf{1}_{2^m\times 1} + \left(\frac{1}{\sigma^2}\right)^2 \mathbf{1}_{1\times n}(\mathbf{\Pi}\#\mathbf{S}\#\mathbf{T})\mathbf{1}_{2^m\times 1} - \left(\frac{1}{\sigma^2}\right)^2 \mathbf{1}_{1\times n}\left\{[(\mathbf{\Pi}\#\mathbf{T})\mathbf{1}_{2^m\times 1}]\#[(\mathbf{\Pi}\#\mathbf{S})\mathbf{1}_{2^m\times 1}]\right\}$$

$$\textbf{(6)}\frac{\partial^2 l(\theta)}{\partial\sigma^2\partial\sigma^2} = \frac{\partial}{\partial\sigma^2}\left[\sum_{i=1}^{n}\sum_{j=1}^{2^m}\frac{1}{\sigma^2}\pi_{ij}\left(\frac{(y_i-\mu_j)^2}{2\sigma^2}-\frac{1}{2}\right)\right]$$
$$= -\left(\frac{1}{\sigma^2}\right)^2\sum_{i=1}^{n}\sum_{j=1}^{2^m}\pi_{ij}(2s_{ij}+\frac{1}{2}) + \frac{1}{\sigma^2}\sum_{i=1}^{n}\sum_{j=1}^{2^m}\frac{\partial\pi_{ij}}{\partial\sigma^2}s_{ij}$$
$$= -\left(\frac{1}{\sigma^2}\right)^2\sum_{i=1}^{n}\sum_{j=1}^{2^m}\pi_{ij}(2s_{ij}+\frac{1}{2}) + \left(\frac{1}{\sigma^2}\right)^2\sum_{i=1}^{n}\sum_{j=1}^{2^m}\pi_{ij}s_{ij}s_{ij}$$
$$-\left(\frac{1}{\sigma^2}\right)^2\sum_{i=1}^{n}\left[\left(\sum_{j=1}^{2^m}\pi_{ij}s_{ij}\right)\left(\sum_{J=1}^{2^m}\pi_{iJ}s_{iJ}\right)\right]$$

In matrix notation, we have:

$$\frac{\partial^2 l(\theta)}{\partial\sigma^2\partial\sigma^2} = -\left(\frac{1}{\sigma^2}\right)^2\mathbf{1}_{1\times n}(\mathbf{\Pi}\#(2\mathbf{S}+\mathbf{H}))\mathbf{1}_{2^m\times 1} + \left(\frac{1}{\sigma^2}\right)^2\mathbf{1}_{1\times n}(\mathbf{\Pi}\#\mathbf{S}\#\mathbf{S})\mathbf{1}_{2^m\times 1}$$
$$-\left(\frac{1}{\sigma^2}\right)^2\mathbf{1}_{1\times n}\left\{[(\mathbf{\Pi}\#\mathbf{S})\mathbf{1}_{2^m\times 1}]\#[(\mathbf{\Pi}\#\mathbf{S})\mathbf{1}_{2^m\times 1}]\right\}$$

where $\mathbf{H}$ is the $n\times 2^m$ matrix where every entry equals $\frac{1}{2}$.

The full log-likelihood second derivative matrix

$$\frac{\partial^2 l(\theta)}{\partial\theta\partial\theta'} = \left(\begin{array}{cccc|c|c}
\frac{\partial^2 l(\theta)}{\partial\theta_1\partial\theta_1} & \cdots & \cdots & \frac{\partial^2 l(\theta)}{\partial\theta_1\partial\theta_c} & \frac{\partial^2 l(\theta)}{\partial\theta_1\partial\mu} & \frac{\partial^2 l(\theta)}{\partial\theta_1\partial\sigma^2} \\
\vdots & \vdots & \vdots & \vdots & \vdots & \vdots \\
\vdots & \vdots & \vdots & \vdots & \vdots & \vdots \\
\frac{\partial^2 l(\theta)}{\partial\theta_c\partial\theta_1} & \cdots & \cdots & \frac{\partial^2 l(\theta)}{\partial\theta_c\partial\theta_c} & \frac{\partial^2 l(\theta)}{\partial\theta_c\partial\mu} & \frac{\partial^2 l(\theta)}{\partial\theta_c\partial\sigma^2} \\
\hline
\frac{\partial^2 l(\theta)}{\partial\mu\partial\theta_1} & \cdots & \cdots & \frac{\partial^2 l(\theta)}{\partial\mu\partial\theta_c} & \frac{\partial^2 l(\theta)}{\partial\mu\partial\mu} & \frac{\partial^2 l(\theta)}{\partial\mu\partial\sigma^2} \\
\hline
\frac{\partial^2 l(\theta)}{\partial\sigma^2\partial\theta_1} & \cdots & \cdots & \frac{\partial^2 l(\theta)}{\partial\sigma^2\partial\theta_c} & \frac{\partial^2 l(\theta)}{\partial\sigma^2\partial\mu} & \frac{\partial^2 l(\theta)}{\partial\sigma^2\partial\sigma^2}
\end{array}\right)$$
$$= \begin{pmatrix} A_{c\times c} & X_{c\times 1} & Z_{c\times 1} \\ X'_{c\times 1} & & \\ & B_{2\times 2} & \\ Z'_{c\times 1} & & \end{pmatrix}$$

where

$$A_{fg} = \frac{\partial^2 l(\theta)}{\partial\theta_f \partial\theta_g} = -\frac{1}{\sigma^2}\mathbf{1}_{1\times n}\mathbf{\Pi}(\mathbf{D}^{[f]}\#\mathbf{D}^{[g]})$$

$$+\left(\frac{1}{\sigma^2}\right)^2 \mathbf{1}_{1\times n}(\mathbf{\Pi}\#\mathbf{T}\#\mathbf{T})(\mathbf{D}^{[f]}\#\mathbf{D}^{[g]}) - \left(\frac{1}{\sigma^2}\right)^2 \mathbf{1}_{1\times n}\left\{[(\mathbf{\Pi}\#\mathbf{T})\mathbf{D}^{[f]}]\#[(\mathbf{\Pi}\#\mathbf{T})\mathbf{D}^{[g]}]\right\}$$

$$X_f = \frac{\partial^2 l(\theta)}{\partial\mu \partial\theta_g} = -\frac{1}{\sigma^2}\mathbf{1}_{1\times n}\mathbf{\Pi}\mathbf{D}^{[g]}$$

$$+\left(\frac{1}{\sigma^2}\right)^2 \mathbf{1}_{1\times n}(\mathbf{\Pi}\#\mathbf{T}\#\mathbf{T})\mathbf{D}^{[g]} - \left(\frac{1}{\sigma^2}\right)^2 \mathbf{1}_{1\times n}\left\{[(\mathbf{\Pi}\#\mathbf{T})\mathbf{D}^{[g]}]\#[(\mathbf{\Pi}\#\mathbf{T})\mathbf{1}_{2^m\times 1}]\right\}$$

$$Z_g = \frac{\partial^2 l(\theta)}{\partial\theta_g \partial\sigma^2} = -\left(\frac{1}{\sigma^2}\right)^2 \mathbf{1}_{1\times n}(\mathbf{\Pi}\#\mathbf{T})\mathbf{D}^{[g]}$$

$$+\left(\frac{1}{\sigma^2}\right)^2 \mathbf{1}_{1\times n}(\mathbf{\Pi}\#\mathbf{S}\#\mathbf{T})\mathbf{D}^{[g]} - \left(\frac{1}{\sigma^2}\right)^2 \mathbf{1}_{1\times n}\left\{[(\mathbf{\Pi}\#\mathbf{T})\mathbf{D}^{[g]}]\#[(\mathbf{\Pi}\#\mathbf{S})\mathbf{1}_{2^m\times 1}]\right\}$$

$$B_{11} = \frac{\partial^2 l(\theta)}{\partial\mu \partial\mu} = -\frac{1}{\sigma^2}\mathbf{1}_{1\times n}\mathbf{\Pi}\mathbf{1}_{2^m\times 1}$$

$$+\left(\frac{1}{\sigma^2}\right)^2 \mathbf{1}_{1\times n}(\mathbf{\Pi}\#\mathbf{T}\#\mathbf{T})\mathbf{1}_{2^m\times 1} - \left(\frac{1}{\sigma^2}\right)^2 \mathbf{1}_{1\times n}\{[(\mathbf{\Pi}\#\mathbf{T})\mathbf{1}_{2^m\times 1}]\#[(\mathbf{\Pi}\#\mathbf{T})\mathbf{1}_{2^m\times 1}]\}$$

$$B_{12} = B_{21} = \frac{\partial^2 l(\theta)}{\partial\mu \partial\sigma^2} = -\left(\frac{1}{\sigma^2}\right)^2 \mathbf{1}_{1\times n}(\mathbf{\Pi}\#\mathbf{T})\mathbf{1}_{2^m\times 1}$$

$$+\left(\frac{1}{\sigma^2}\right)^2 \mathbf{1}_{1\times n}(\mathbf{\Pi}\#\mathbf{S}\#\mathbf{T})\mathbf{1}_{2^m\times 1} - \left(\frac{1}{\sigma^2}\right)^2 \mathbf{1}_{1\times n}\{[(\mathbf{\Pi}\#\mathbf{T})\mathbf{1}_{2^m\times 1}]\#[(\mathbf{\Pi}\#\mathbf{S})\mathbf{1}_{2^m\times 1}]\}$$

$$B_{22} = \frac{\partial^2 l(\theta)}{\partial\sigma^2 \partial\sigma^2} = -\left(\frac{1}{\sigma^2}\right)^2 \mathbf{1}_{1\times n}(\mathbf{\Pi}\#(2\mathbf{S}+\mathbf{H}))\mathbf{1}_{2^m\times 1}$$

$$+\left(\frac{1}{\sigma^2}\right)^2 \mathbf{1}_{1\times n}(\mathbf{\Pi}\#\mathbf{S}\#\mathbf{S})\mathbf{1}_{2^m\times 1} - \left(\frac{1}{\sigma^2}\right)^2 \mathbf{1}_{1\times n}\{[(\mathbf{\Pi}\#\mathbf{S})\mathbf{1}_{2^m\times 1}]\#[(\mathbf{\Pi}\#\mathbf{S})\mathbf{1}_{2^m\times 1}]\}$$

Note that the first term in B_{11}, namely $-\frac{1}{\sigma^2}\mathbf{1}_{1\times n}\mathbf{\Pi}\mathbf{1}_{2^m\times 1}$ equals $-\frac{1}{\sigma^2}n$.

References

1. Kao CH, Zeng ZB, Teasdale RD (1999) Multiple interval mapping for quantitative trait loci. Genetics 152: 1203–1216.

2. Zou F, Fine JP, Hu J, Lin DY (2004) An efficient resampling method for assessing genome-wide statistical significance in mapping quantitative trait loci. Genetics 168: 2307–2316.
